# Supplementary material for: Dynamic transcriptomic profiles of zebrafish gills in response to zinc supplementation
Source: BMC Genomics. 2010 Oct 11;11:553. doi: 10.1186/1471-2164-11-553 (PMC3091702; doi:10.1186/1471-2164-11-553)
Supplement: Additional file 2 — Interactive Direct Interaction Network representing the molecular interactions between zinc, copper, iron, calcium and proteins encoded by transcripts changed by zinc supplementation. Mini web-site containing index.html and hyperlinked pages in subdirectory describing a Direct Interaction Network automatically generated based on curated interactions contained within the proprietary PathwayArchitect database. Ovals represent proteins and the circles symbolize metal ions. Objects are coloured by their abundance in zebrafish at the time-point they were significantly different from the control is a scale from -4 fold (dark green) to +4 fold (dark red). Where significant differences were found at more than one time-point, the colour overlay shows expression at the first instance. Dark blue squares denote 'binding', and light blue squares 'expression'; green squares stand for 'regulation', green diamonds for 'metabolism', and green circles for 'promoter binding'. Arrow heads indicate directionality of the interaction where annotated. All nodes and edges can be further interrogated by selecting the relative area of the image. [file 1471-2164-11-553-S2.zip › PathwayArchitect Zn xs DIN/95334.html]

# PROTEIN: ZP3

|  |  |
| --- | --- |
| Name | ZP3 |
| Type | PROTEIN |
| Description | zona pellucida glycoprotein 3 (sperm receptor) |
| Note | The zona pellucida is an extracellular matrix that surrounds the oocyte and early embryo. It is composed primarily of three or four glycoproteins with various functions during fertilization and preimplantation development. The protein encoded by this gene is a structural component of the zona pellucida and functions in primary binding and induction of the sperm acrosome reaction. The nascent protein contains a N-terminal signal peptide sequence, a conserved ZP domain, a C-terminal consensus furin cleavage site, and a transmembrane domain. It is hypothesized that furin cleavage results in release of the mature protein from the plasma membrane for subsequent incorporation into the zona pellucida matrix. However, the requirement for furin cleavage in this process remains controversial based on mouse studies. A variation in the last exon of this gene has previously served as the basis for an additional ZP3 locus; however, sequence and literature review reveals that there is only one full-length ZP3 locus in the human genome. Another locus encoding a bipartite transcript designated POMZP3 contains a duplication of the last four exons of ZP3, including the above described variation, and maps closely to this gene. |
| Alias | ZPC |
|  | Zp-3 |
|  | zona pellucida protein C |
|  | ZP3 |
|  | zona pellucida glycoprotein 3B |
|  | zona pellucida glycoprotein 3A (sperm receptor) |
|  | Sperm receptor |
|  | HUMZP3 |
|  | zona pellucida glycoprotein 3A |
|  | Zpc |
|  | Zp3 |
|  | zona pellucida glycoprotein 3 |
|  | sperm receptor |
|  | Zona pellucida glycoprotein ZP3 |
|  | ZP3A/ZP3B |
|  | Zona pellucida glycoprotein 3 |
|  | ZP3-424 |
|  | ZP3-372 |
|  | Zona pellucida protein C |
|  | ZP3B |
|  | zona pellucida sperm-binding protein 3 precursor |
|  | ZP3A |


---

|  |  |
| --- | --- |
| GO Component | extracellular space |
|  | integral to membrane |
|  | extracellular matrix (sensu Metazoa) |


---

|  |  |
| --- | --- |
| GO ID | GO:0005615 |
|  | GO:0005515 |
|  | GO:0004872 |
|  | GO:0016021 |
|  | GO:0007338 |
|  | GO:0005102 |
|  | GO:0015026 |
|  | GO:0005578 |
|  | GO:0007339 |


---

|  |  |
| --- | --- |
| MIM | MIM:182889 |


---

|  |  |
| --- | --- |
| Connectivity | 63 |


---

|  |  |
| --- | --- |
| Entrez ID | 22788 |
|  | 7784 |
|  | 114639 |


---

|  |  |
| --- | --- |
| Agilent ID | A\_24\_P95059 |
|  | A\_14\_P129552 |
|  | A\_23\_P134419 |
|  | A\_52\_P42762 |
|  | A\_44\_P320669 |
|  | A\_43\_P12925 |
|  | A\_51\_P152977 |
|  | A\_51\_P152973 |
|  | A\_14\_P132234 |
|  | A\_53\_P161772 |


---

|  |  |
| --- | --- |
| Cellular Localization | Extracellular matrix |
|  | Extracellular region |
|  | Membrane |
|  | Cell |


---

|  |  |
| --- | --- |
| Pathway | Zn xs inventory |
|  | Zn xs DIN |


---

|  |  |
| --- | --- |
| GO Process | binding of sperm to zona pellucida |
|  | fertilization (sensu Metazoa) |


---

|  |  |
| --- | --- |
| UniGene | Hs.130553 |
|  | Hs.488877 |
|  | Rn.10892 |
|  | Mm.1381 |


---

|  |  |
| --- | --- |
| Affymetrix Probeset ID | 102675\_at |
|  | 1368757\_at |
|  | 1419007\_at |
|  | 204148\_s\_at |
|  | x14376\_s\_at |
|  | Y10823cds\_s\_at |
|  | 39719\_at |
|  | 39720\_g\_at |
|  | 74581\_at |
|  | U10099\_s\_at |
|  | g12653432\_3p\_s\_at |


---

|  |  |
| --- | --- |
| GO Function | protein binding |
|  | receptor activity |
|  | receptor binding |
|  | coreceptor activity |


---

|  |  |
| --- | --- |
| Nucleotide | AY057778 |
|  | AY057779 |
|  | AY057764 |
|  | AY057776 |
|  | BC099465 |
|  | NM\_053762 |
|  | M60504 |
|  | Y10823 |
|  | BC103585 |
|  | AY057762 |
|  | X14376 |
|  | AK131934 |
|  | AY057763 |
|  | M20026 |
|  | NM\_007155 |
|  | D78482 |
|  | AY057761 |
|  | BC103583 |
|  | AY057748 |
|  | AK056788 |
|  | CA421411 |
|  | AY057777 |
|  | BC100745 |
|  | AY057747 |
|  | AY057746 |
|  | BC103584 |
|  | X56777 |
|  | BI828655 |
|  | A18567 |
|  | NM\_011776 |
|  | AY057749 |


---

|  |  |
| --- | --- |
| Protein | AAL23765 |
|  | NP\_009086 |
|  | AAL23780 |
|  | CAA40095 |
|  | AAL23768 |
|  | NP\_446214 |
|  | CAA71787 |
|  | AAL23783 |
|  | AAB18629 |
|  | AAL23782 |
|  | CAA32550 |
|  | AAI03584 |
|  | AAH99465 |
|  | AAI03585 |
|  | CAA01398 |
|  | P97708 |
|  | AAL23796 |
|  | AAL23795 |
|  | P21754 |
|  | AAI00746 |
|  | P10761 |
|  | AAA61336 |
|  | BAA24456 |
|  | AAL23767 |
|  | AAL23798 |
|  | AAL23766 |
|  | NP\_035906 |
|  | AAL23797 |
|  | AAI03586 |
|  | BAE20883 |
|  | AAL23781 |


---

|  |  |
| --- | --- |
| Organism | Mammal |


---

|  |  |
| --- | --- |
| Location | chromosome 5, 5 77.0 cM, 5 G2 (Mus musculus) |
|  | 5 77.0 cM (Mus musculus) |
|  | chromosome 7, 7q11.23 (Homo sapiens) |
|  | chromosome 12, 12q12 (Rattus norvegicus) |


---

|  |  |
| --- | --- |
